# Combinatorial Effects of Plasma-Treated Solution and Plasma-Treated Hydrogel with Cisplatin in Vulvar Cancer Cells

**DOI:** 10.32604/or.2026.081755

**Published:** 2026-08-13

**Authors:** Estelle C. I. D. Schad, Janet P. Raja Xavier, Hortense Decool, Marcel Arnholdt, Franziska Keßler, Jan Schöttke, Sara Y. Brucker, Ernst Oberlechner, Johanna Laupp, Martin Weiss

**Affiliations:** 1 Department of Women’s Health Tübingen, University of Tübingen, Tübingen, Germany; 2 Natural and Medical Sciences Institute (NMI), University of Tübingen, Reutlingen, Germany

**Keywords:** Vulvar squamous cell carcinoma, low-thermal argon plasma devitalization (ltAPD), non-invasive physical plasma (NIPP), cold atmospheric plasma (CAP), reactive oxygen and nitrogen species, combination therapy

## Abstract

**Background:** Vulvar squamous cell carcinoma (VSCC) is a rare, but increasingly prevalent malignancy with limited therapeutic options in the advanced disease state. Cisplatin-based chemoradiation remains the standard of care but is constrained by cumulative toxicity. Precancerous lesions, including high-grade squamous intraepithelial lesions (HSIL) and differentiated vulvar intraepithelial neoplasia (dVIN), similarly require effective yet tolerable treatments. Low-thermal Argon plasma devitalization (ltAPD), a source of reactive oxygen and nitrogen species (RONS), has emerged as a promising approach for redox-based tumor modulation. This study aimed to evaluate the anti-tumor efficacy of two plasma modalities, plasma-treated solution (PTS) and plasma-treated hydrogel (PTH) in combination with cisplatin in VSCC cells. **Methods:** PTS and PTH were applied as monotherapies and in sequential combination with cisplatin (plasma-first vs. cisplatin-first). RONS kinetics, cell viability, and treatment interactions were assessed following plasma monotherapy and sequential combination with cisplatin. Apoptosis and redox balance were evaluated by caspase-3/7 activity and GSH/GSSG ratios, respectively. **Results:** PTS induced a rapid oxidative burst, whereas PTH enabled delayed and sustained RONS release. Both modalities reduced cell viability in a time-dependent manner, with rapid cytotoxic effects observed for PTS. Plasma pre-treatment enhanced cisplatin efficacy, with higher synergy scores in the plasma-first sequence. Increased caspase-3/7 activity and decreased GSH/GSSG ratios, observed in the combination treatment of plasma and cisplatin, indicated enhanced oxidative stress and apoptotic priming. **Conclusions:** PTS and PTH exhibit intrinsic anti-tumor activity and sensitize VSCC cells to cisplatin through redox modulation. These findings support the integration of plasma-based strategies into combinatorial therapeutic approaches for VSCC and its precancerous lesions.

## 1 Introduction

Vulvar cancer is the fourth most common gynecological malignancy worldwide, accounting for approximately 4% of all cancers affecting the female genital tract [1]. Although relatively rare, its incidence is steadily increasing, with the mean age of incidence falling [1,2]. The majority of vulvar cancers are squamous cell carcinomas (VSCC), while other less common types include vulvar melanoma, basal cell carcinoma (BCC), and Paget’s disease. Many cases of VSCC arise from precancerous lesions, which are classified based on their association with human papillomavirus (HPV) [3,4]. Low-grade squamous intraepithelial lesions (LSIL), also known as vulvar intraepithelial neoplasia (VIN) I, are typically linked to low-risk HPV type infections and have low malignant potential [5]. In contrast, high-grade squamous intraepithelial lesions (HSIL), also classified as VIN II and VIN III, are associated with persistent HPV infections and carry a higher risk of malignant progression [4,5]. Differentiated VIN (dVIN) is an HPV-independent precursor lesion, often related to chronic inflammatory conditions such as lichen sclerosus, and is known for its aggressive progression to invasive VSCC [6].

The treatment of vulvar cancer typically involves a combination of surgery and chemoradiotherapy, with treatment decisions based on disease stage, metastatic spread, and recurrence risk [4,7,8]. Surgical options range from local excision for early-stage tumors to radical vulvectomy for more extensive disease, often accompanied by lymph node assessment [3,9]. Chemoradiotherapy serves multiple roles, including neoadjuvant therapy to shrink tumors before surgery, adjuvant therapy to reduce recurrence risk, definitive therapy for inoperable cases, and palliative care to manage symptoms in advanced stages [10,11]. These interventions, however, have significant limitations. Radical surgical procedures are often associated with physical and psychological distress, including disfigurement, chronic pain, and wound healing complications [12]. While cisplatin-based chemoradiation can achieve complete response rates of 87%, effective tumor control often requires high cumulative doses, and treatment-related toxicity frequently represents the limiting factor of current therapeutic approaches [10]. The development of chemoresistance additionally reduces long-term efficacy. Additionally, treatment options for advanced or recurrent disease remain scarce, with high recurrence rates and a lack of effective targeted therapies. Therefore, there is a critical need for novel, less invasive, more precise, and better-tolerated treatment strategies to improve patient outcomes and quality of life. In particular, additive therapeutic approaches are warranted that can enhance or maintain treatment efficacy while reducing the cumulative toxicity burden of current multimodal regimens.

Low-thermal argon plasma devitalization (ltAPD) has recently emerged as a novel cancer therapy that activates and enhances anti-tumor responses through diverse mechanisms [13]. ltAPD uses a partially ionized gas, generated at room temperature and atmospheric pressure, containing various short-, intermediate- and long-living reactive oxygen and nitrogen species (RONS) [13,14]. RONS function as pleiotropic signaling molecules within cells, and at elevated levels, they can induce detrimental effects, including pathophysiological responses such as cell death [14,15]. Advances in plasma medicine have enabled the precise generation and targeted delivery of controlled RONS combinations, allowing ltAPD to target critical processes in tumor progression with effective therapeutic strategies [16,17,18].

The two primary ltAPD treatment modalities are direct and indirect applications [19]. Direct ltAPD treatment delivers plasma directly to cancer cells or tissues, offering high precision for superficial tumors or precancerous lesions previously shown in several prospective clinical trials on the treatment of pre-cancerous cervical lesions [20,21,22,23,24]. Indirect ltAPD treatment generates plasma-treated solutions (PTS) by enriching a liquid medium with RONS, which can then be injected or perfused to reach deeper tumors [19,24]. However, PTS present challenges in maintaining controlled RONS delivery, as they are often diluted by bodily fluids upon administration. To this end, plasma-treated hydrogels (PTHs) are shown to offer a novel, hybrid form of direct and indirect ltAPD treatment, capable of extending the stability and therapeutic delivery of plasma-derived RONS [25].

A growing body of evidence indicates that ltAPD renders enhanced anti-cancer efficacy when used in combination with conventional chemotherapeutic agents [26,27]. ltAPD as a potential combinatorial treatment to mitigate tumor burden and overcome drug resistance in cancer is a promising area of research, with the potential to improve the effectiveness of existing anticancer drugs, lower overall cytotoxicity, and develop new therapeutic strategies. Among these, cisplatin, a platinum-based drug widely employed in the treatment of gynecological cancers, represents an interesting candidate for plasma-mediated combination therapy [26,27]. Cisplatin, commonly administered concurrently with radiotherapy, exerts its cytotoxic effects primarily through the formation of DNA interstrand crosslinks and the induction of oxidative stress; however, its clinical utility is frequently limited by dose-dependent systemic toxicity and the development of chemoresistance [28]. Preclinical studies in other tumor models suggest that ltAPD synergies with cisplatin, reinforces DNA damage, increasing intracellular reactive oxygen species (ROS) accumulation and modulates drug transport, thus enhancing or restoring sensitivity in resistant cell populations [26,27,29].

The present study aims to study the anti-cancer potential of PTS and PTH in an *in vitro* VSCC model to evaluate their synergistic efficacy when combined with cisplatin. Particularly, this work explores the ability of plasma-based therapies to potentiate cisplatin cytotoxicity, disrupt redox balance, while comparing the effectiveness of PTS versus PTH as plasma delivery modalities. By incorporating plasma-based approaches with conventional chemotherapy, this study seeks to establish a preclinical rationale for combinatorial treatment strategies that are less invasive, more effective, and translatable for clinical management of vulvar cancer.

## 2 Materials and Methods

### 2.1 Cell Line and Culture Conditions

Human vulvar carcinoma SW954 cells (American Type Culture Collection, #HTB-117™, Manassas, VA, USA) were cultured at 37°C in a humidified atmosphere containing 5% CO_2_ in Dulbecco’s Modified Eagle’s Medium (DMEM) (Gibco, #41965-039, Paisley, Scotland, UK) supplemented with 10% (*v*/*v*) fetal bovine serum (FBS) (Gibco, #10270-106) and 1% (*v*/*v*) antibiotic-antimycotic solution (Gibco, #15140-122). According to the supplier, the cell line is authenticated by short tandem repeat (STR) profiling. ATCC certifies that all distributed cell lines are free of mycoplasma contamination at the time of shipment. Cells were cultured and maintained under sterile conditions in accordance with the supplier’s instructions. Cells were detached using 0.05% Trypsin-EDTA (Gibco, #25300-062) and seeded at densities of 2 × 10^4^ cells per well in 96-well plates and cultured to 80% confluence.

### 2.2 Plasma Source

ltAPD was generated using a 3.2 mm flexible probe (FiAPC^®^ 2200a, 3.2 mm diameter) connected to a RF-power generator (VIO^®^3) and an APC 3 module (both Erbe Elektromedizin GmbH, Germany) operated in precise APC E1 mode with an argon gas flow of 1.6 L/min. The setup used (Table 1) is well-established considering the treatment of human tissue in open-surgical or endoscopic/laparoscopic applications.

**Table 1:** Overview of the plasma treatment parameters applied in this study.

Parameter	Experimental Condition
Peak Voltage (V_peak_)	4.5 kV
Waveform	Damped sinusoidal
Carrier Frequency (f_c_)	350 kHz
Modulation Frequency (f_m_)	20 kHz
Average Power (P_avg_)	2 W

### 2.3 Generation and Application of Plasma-Treated Solution (PTS)

PTS was generated by exposing 1 mL of serum-free DMEM (Gibco, #41965-039) to ltAPD in a metal container placed on the neutral plate electrode. The distance between the probe and the liquid surface was maintained manually at 5 mm, using a mm scale. Nevertheless, possible deviations fall within a range of 4–8 mm from the liquid. 24 h after cell seeding (Section 2.1), the culture medium was replaced with 100 μL of freshly generated PTS, which was immediately applied after generation. Cells were subsequently incubated under standard culture conditions. A plasma-free control group (0 s PTS), consisting of serum-free DMEM (Gibco, #41965-039), was included throughout the cell culture experiments.

### 2.4 Generation and Application of Plasma-Treated Hydrogel (PTH)

For PTH preparation, *in vitro* and *in vivo* [30,31] established potato starch-based powdered 4DryField^®^ PH powder (PlantTec Medical, Lüneburg, Niedersachsen, Germany) was used. To generate PTH, 4DryField^®^ PH was mixed with PTS at a w/v ratio of 0.08 g/mL (e.g., 0.03 g of powder with 375 μL of PTS). A 0 s PTH condition served as the non-plasma-treated hydrogel control and consisted of serum-free DMEM (Gibco, #41965-039) mixed with 4DryField^®^ PH powder. Samples comprising a combination of PTS and 4DryField^®^ PH exhibited immediate hydrogel formation, resulting in a viscous gel. For treatments, the culture medium was removed from the adherent cells, and a total volume of 50 μL PTH was transferred covering the whole area of a 96-well. Then, each well was covered with 50 μL serum-free DMEM (Gibco, #41965-039) before incubation. After 24 h of application, PTH was diluted with 100 μL of DMEM (Gibco, #41965-039), subsequently removed and washed again with 100 μL of DMEM (Gibco, #41965-039) to remove the residuals of the gel. Subsequently 100 μL of DMEM (Gibco, #41965-039) were applied and further assays were performed.

### 2.5 Cisplatin Treatment

Cisplatin (MedChemExpress, #HY-17394, Monmouth Junction, NJ, USA) was dissolved in previously introduced (Section 2.1) FBS-supplemented DMEM (Gibco, #41965-039) and applied at final concentrations of 3, 6, 12, 24, 48, and 96 μM for 2, 4, 6 and 24 h.

### 2.6 Combinatorial Plasma Treatment with Cisplatin

To evaluate the sequence-dependent effects of plasma-treated modalities and cisplatin, two combinatorial treatment modalities (PTS and PTH), each combined with cisplatin, were applied to the vulvar carcinoma cell line SW954 following the subsequent protocol (*Sequence 1* and *Sequence 2*).

*Sequence 1: Cisplatin → PTS/PTH.*

Cells were first exposed to cisplatin at the indicated concentrations (12, 24, 48, 72, and 96 μM) for 2 h under standard culture conditions (37°C, 5% CO_2_). Following this pre-treatment period, the cisplatin-containing medium was aspirated to stop drug exposure and minimize residual cytotoxic effects. Cells were then immediately treated with freshly prepared PTS (0, 30, 60, and 90 s) or PTH (0, 30, 60, 90, 120, and 180 s) and incubated for an additional 24 h.

*Sequence 2: PTS/PTH → Cisplatin.*

In the reverse treatment sequence, cells were initially exposed to PTS (0, 30, 60, and 90 s) or PTH (0, 30, 60, 90, 120, and 180 s) for 2 h to induce redox modulation and potential oxidative priming. After this incubation period, the plasma-treated medium or hydrogel was removed, and cells were washed where appropriate and subsequently exposed to cisplatin at the indicated concentrations (12, 24, 48, 72, and 96 μM) for 24 h.

Control groups included untreated cells (0 s PTS/PTH and 0 μM cisplatin), cisplatin-only treated cells (0 s PTS/PTH), and PTS or PTH only treated cells (0 μM cisplatin) to distinguish combinatorial effects from monotherapy responses.

Following the respective treatment sequences, cell viability was assessed using an MTS assay (Section 2.8). The obtained viability data were used to evaluate potential synergistic effects between cisplatin and the plasma treatment modalities (PTS or PTH) (Section 2.11) and to assess the statistical significance of the combined treatment compared to the respective controls (Section 2.12).

### 2.7 Quantification of RONS and pH

H_2_O_2_, NO_2_^−^, and pH were measured in PTS and in medium from PTH-treated wells using semi-quantitative test strips (Macherey-Nagel, #91319 for H_2_O_2_; #91313 for NO_2_^−^, Dueren, Nordrhein-Westfalen, Germany) (Carl Roth, #0549.2 for pH, Karlsruhe, Baden-Wuerttemberg, Germany). Strips were analyzed with a Quantofix Relax strip reader (Macherey-Nagel, #91346), and concentrations were recorded immediately after treatment (0 h) and following defined incubation times (1, 2, 4, 6, 8, 24, 48, and 72 h). PTS and PTH treatment times of 1, 3, and 5 min were evaluated.

### 2.8 Cell Viability Assay

Cell viability was assessed using the MTS assay (Promega Corporation, #G3582, Madison, WI, USA) according to the manufacturer’s instructions. After the performed treatments (Section 2.3, Section 2.4, Section 2.5 and Section 2.6), the treatment medium was replaced with fresh medium. Specifically, for PTH treatments, the washing steps described in Section 2.4 were performed to remove residuals of the gel, before covering the SW954 cells with fresh DMEM (Gibco, #41965-039). Subsequently, 15 μL of MTS reagent (Promega Corporation, #G3582) were mixed with 15 μL Dulbecco’s Phosphate Buffered Saline (DPBS) (PAN-Biotech, #P04-36500, Aidenbach, Bavaria, Germany) in a 50% (*v*/*v*) ratio. The 30 μL mix was added to each well containing 100 μL of DMEM (Gibco, #41965-039), resulting in a 30% (*v*/*v*) ratio. After incubation for 2 h, absorbance was measured at 490 nm using the Varioskan LUX Multimode Microplate Reader (Thermo Fisher Scientific, Waltham, MA, USA). Background correction was performed using cell-free DMEM (Gibco, #41965-039) blanks. For each tested condition, three biological replicates consisting of three technical replicates were performed. Cell viability was normalized to the 0 s PTS/PTH combined with 0 μM cisplatin control, which served as the 100% reference value.

### 2.9 Caspase-3/7 Activity

Caspase-3/7 activation was quantified using the Caspase-Glo^®^ 3/7 assay (Promega, #G8091, Madison, WI, USA) as per the manufacturer’s instructions. SW954 cells were subjected to the previously introduced combination protocols in Section 2.6. Distinctly different endpoints were used: Notably for sequence 1, cisplatin concentrations of 0, 12, 48, and 72 μM were applied for 2 h, followed by a 6 h PTS (0, 30, and 60 s) or PTH (0, 30, and 60 s) treatment. For sequence 2, PTS (0, 30, and 60 s) or PTH (0, 30, and 60 s) was applied for 2 h, followed by a 6 h cisplatin treatment (0, 12, 48, and 72 μM). After treatment, SW954 cells were equilibrated to room temperature. 100 μL Caspase-Glo^®^ reagent as supplied in the kit (Promega, #G8091) was subsequently applied to each well, resulting in a total volume of 200 μL per well (50% *v*/*v*). Plates were shaken briefly and incubated at room temperature for 1 h before luminescence recording using the Varioskan LUX Multimode Microplate Reader (Thermo Fisher Scientific).

### 2.10 Glutathione Redox Status (GSH/GSSG)

Intracellular GSH and GSSG levels were measured using the GSH/GSSG-Glo™ assay (Promega, #V6611), following the supplier’s protocol. SW954 cells were subjected to the previously introduced combination protocols in Section 2.6. For sequence 1, cisplatin concentrations of 0 μM and 72 μM were applied for 2 h, followed by a 24 h PTS (0, 30, and 60 s) or PTH (0, 30, and 60 s) treatment. For sequence 2, PTS (0, 30, and 60 s) or PTH (0, 30, and 60 s) was applied for 2 h, followed by a 24 h cisplatin treatment (0 μM and 72 μM). Luminescence was recorded on the Varioskan LUX Multimode Microplate Reader (Thermo Fisher Scientific). For each biological replicate, a standard curve was generated using defined GSH concentrations (0.25–16 μM), allowing conversion of luminescence (in RLU) to GSH and GSSG concentrations as instructed by the manufacturer. GSH/GSSG ratios were subsequently calculated from the GSH standard curve strictly following the manufacturer’s instructions as the quotient of reduced GSH to oxidized GSSG concentrations, accounting for the 2:1 stoichiometry between GSH and GSSG.

### 2.11 Synergy Analysis

Synergy scores were computed using the SynergyFinder+ web application (https://synergyfinder.org/, accessed on 03 August 2025) [32,33] based on the Zero Interaction Potency (ZIP) model. For each combination experiment described in Section 2.6, each tested cisplatin concentration (0, 12, 24, 48, 72, and 96 μM) was combined with each plasma treatment condition to generate full dose–response matrices. For PTS, cisplatin was combined with treatment durations of 0, 30, 60, and 90 s, yielding a 6 × 4 matrix; for PTH, cisplatin was combined with treatment durations of 0, 30, 60, 90, 120, and 180 s, yielding a 6 × 6 matrix. Cell viability was normalized to the untreated control (0 μM cisplatin and 0 s PTS/PTH). For each cisplatin–plasma treatment combination, the mean viability of three biological replicates was calculated and used as input for the ZIP model analysis. To avoid overinterpretation of minor interaction effects, a conservative threshold was applied, with ZIP scores >20 classified as synergistic and <–20 as antagonistic, while scores around 0 indicate no interaction between the compared treatments. No additional statistical significance testing of the synergy scores was performed.

### 2.12 Statistical Analysis

**Figure 1:**
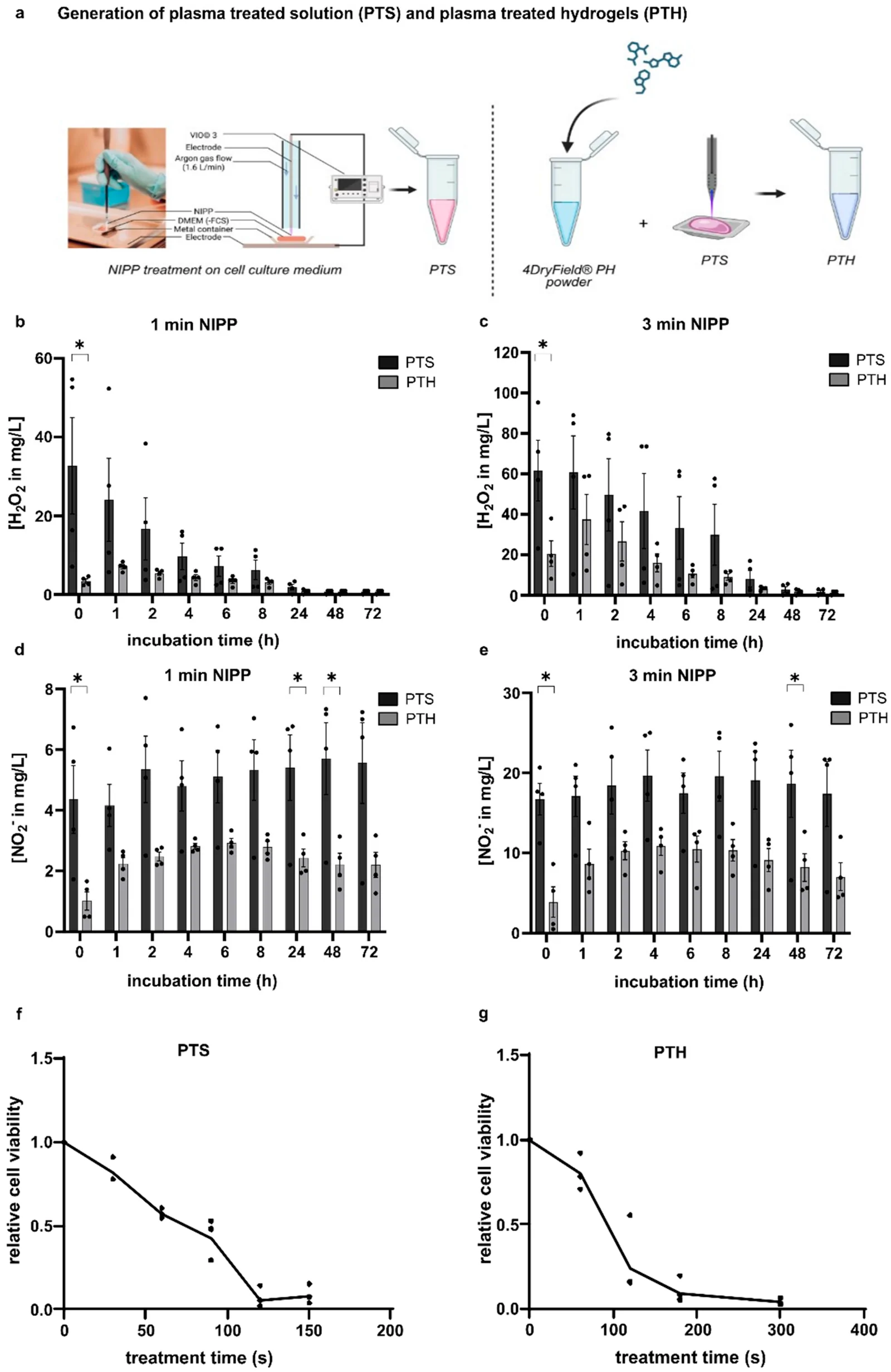
**RONS dynamics in Plasma Treated Solution (PTS) and Plasma Treated Hydrogel (PTH) treatment modalities.** (**a**) Schematic representation of the generation of PTS and PTH using low-thermal argon plasma devitalization (ltAPD). (**b**) Hydrogen peroxide (H_2_O_2_) concentrations were measured immediately after plasma treatment (0 h) and following incubation periods ranging from 1–72 h for 1 min of plasma treatment. (**c**) Hydrogen peroxide (H_2_O_2_) concentrations were measured immediately after plasma treatment (0 h) and following incubation periods ranging from 1–72 h for 3 min of plasma treatment. (**d**) Nitrite (NO_2_^−^) concentrations were measured immediately after treatment (0 h) and after incubation for 1–72 h for 1 min of plasma treatment. (**e**) Nitrite (NO_2_^−^) concentrations were measured immediately after treatment (0 h) and after incubation for 1–72 h for 3 min of plasma treatment. (**f**) Relative cell viability of SW954 cells treated with PTS at varied plasma activation time (s). (**g**) Relative cell viability of SW954 cells treated with PTH at varied plasma activation time (s). Data are presented as mean ± SEM, *p < 0.05. Each data point represents one biological replicate.

Statistical analyses were performed using GraphPad Prism 9 (GraphPad Software, San Diego, CA, USA). For longitudinal RONS measurements (Fig. 1), statistical significance was evaluated using a two-way repeated-measures ANOVA, followed by Šídák multiple comparison test to compare PTS and PTH after each incubation time point. For the pH measurements, a two-way repeated measures ANOVA, followed by Dunnett’s multiple comparison test, was used to evaluate statistical significances within PTS and PTH across increasing incubation times compared to the 0 h time point.

**Figure 2:**
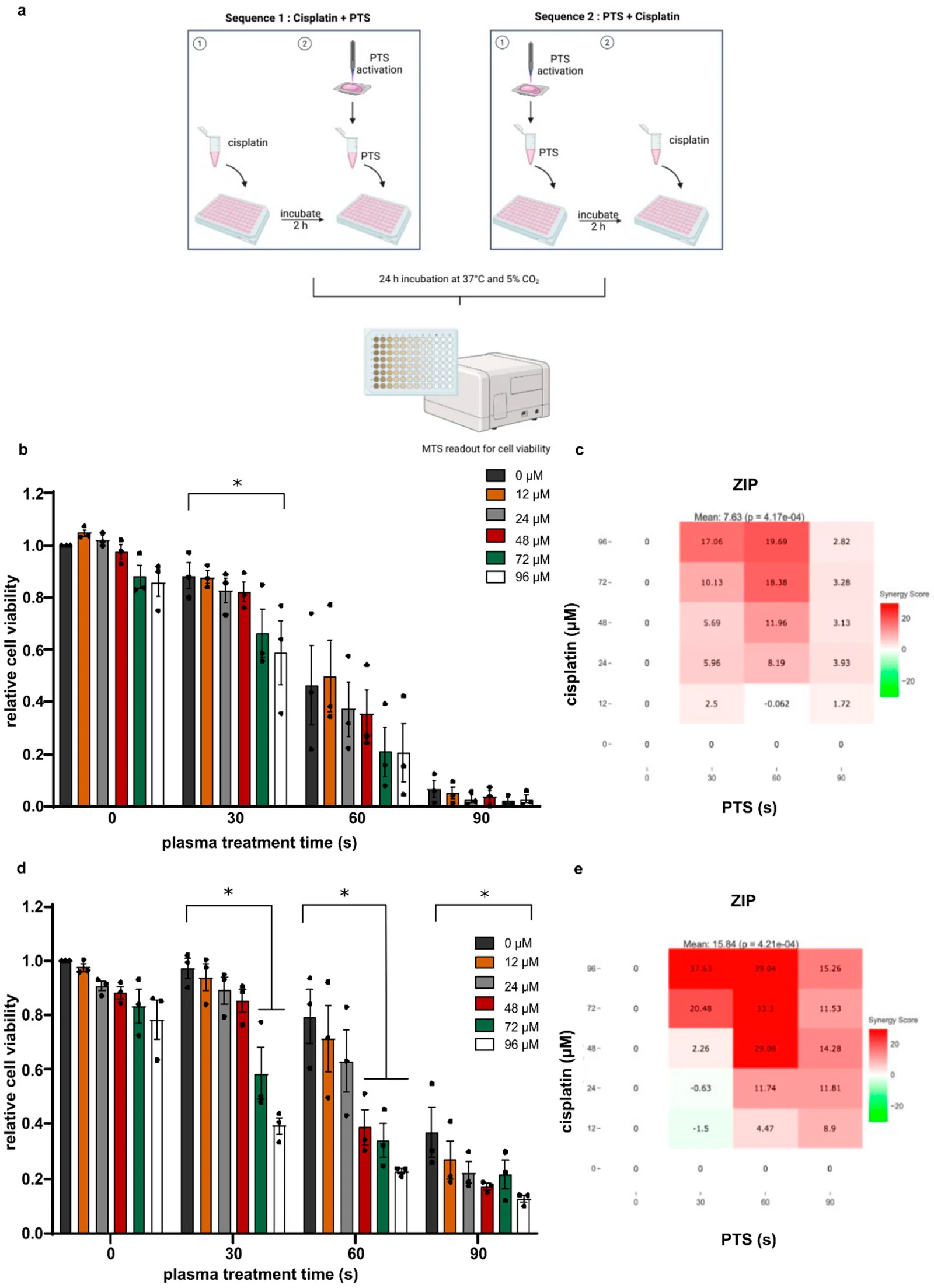
**Cytotoxicity and synergy of cisplatin combined with PTS.** (**a**) Schematic illustration of the experimental setup of both tested treatment sequences with plasma and cisplatin treatment combinations. (**b**) Dose-response curve was generated with the first sequence, cell viability of SW954 treated with a 2 h cisplatin pre-treatment followed by a 24 h PTS treatment. (**c**) Calculated synergy score for sequence 1 (cisplatin → PTS). (**d**) Cell viability of SW954 treated with a 2 h PTS pre-treatment followed by a 24 h cisplatin treatment. (**e**) The calculated synergy score for sequence 2 (PTS → cisplatin). Data are shown as the mean ± SEM, *p < 0.05. Each data point represents one biological replicate.

**Figure 3:**
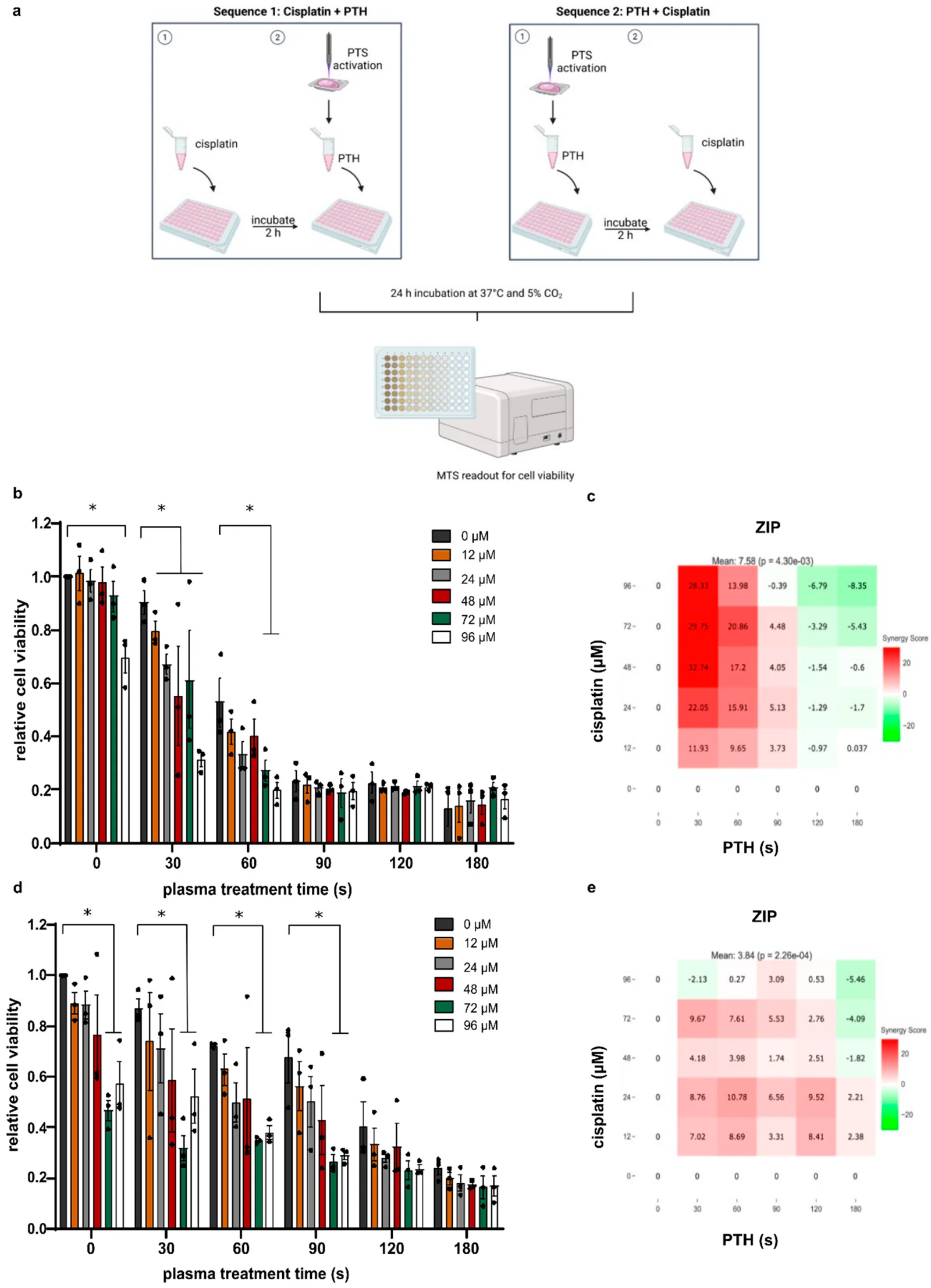
**Cytotoxicity and synergy of cisplatin combined with PTH.** (**a**) Schematic representation of experimental setup of both tested treatment sequences with cisplatin and PTH. (**b**) Cell viability of SW954 treated with a 2 h cisplatin pre-treatment followed by a 24 h PTH treatment. (**c**) Calculated synergy score for sequence 1 (cisplatin → PTH). (**d**) Cell viability of SW954 treated with a 2 h PTH pre-treatment followed by a 24 h cisplatin treatment. (**e**) The calculated synergy score for sequence 2 (PTH → cisplatin). Data are shown as the mean ± SEM, *p < 0.05. Each data point represents one biological replicate.

**Figure 4:**
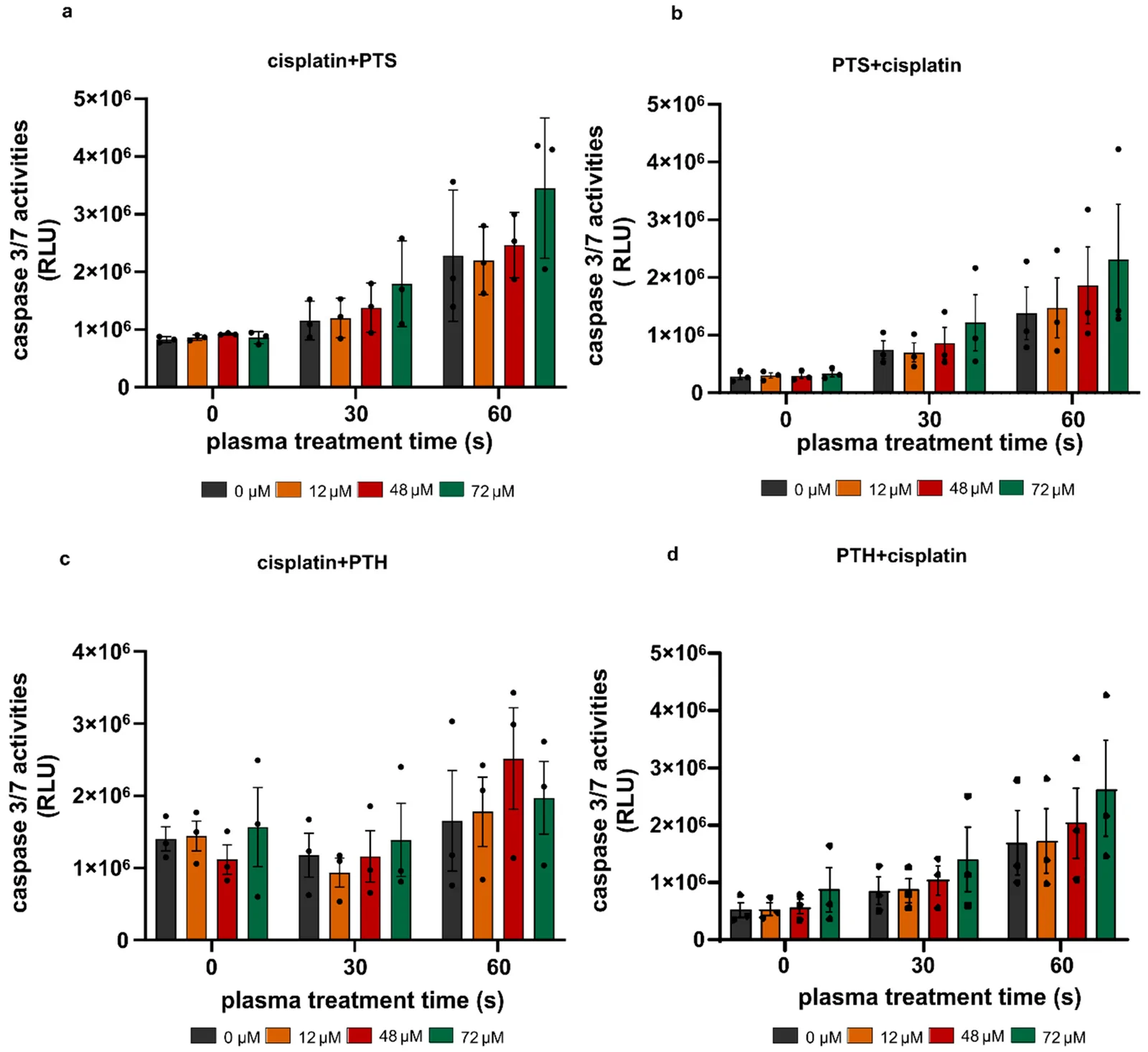
**Caspase-3/7 activation following plasma mediated combinations treatment with cisplatin.** Both treatment sequences were evaluated with PTS and PTH. (**a**) Caspase-3/7 activity following cisplatin pre-treatment for 2 h followed by 6 h of PTS. (**b**) Caspase-3/7 activity following PTS pre-treatment for 2 h followed by 6 h cisplatin treatment. (**c**) Caspase-3/7 activity following cisplatin pre-treatment for 2 h followed by 6 h PTH. (**d**) Caspase-3/7 activity following PTH pre-treatment for 2 h followed by 6 h cisplatin. Data are shown as the mean ± SEM. Each data point represents one biological replicate.

**Figure 5:**
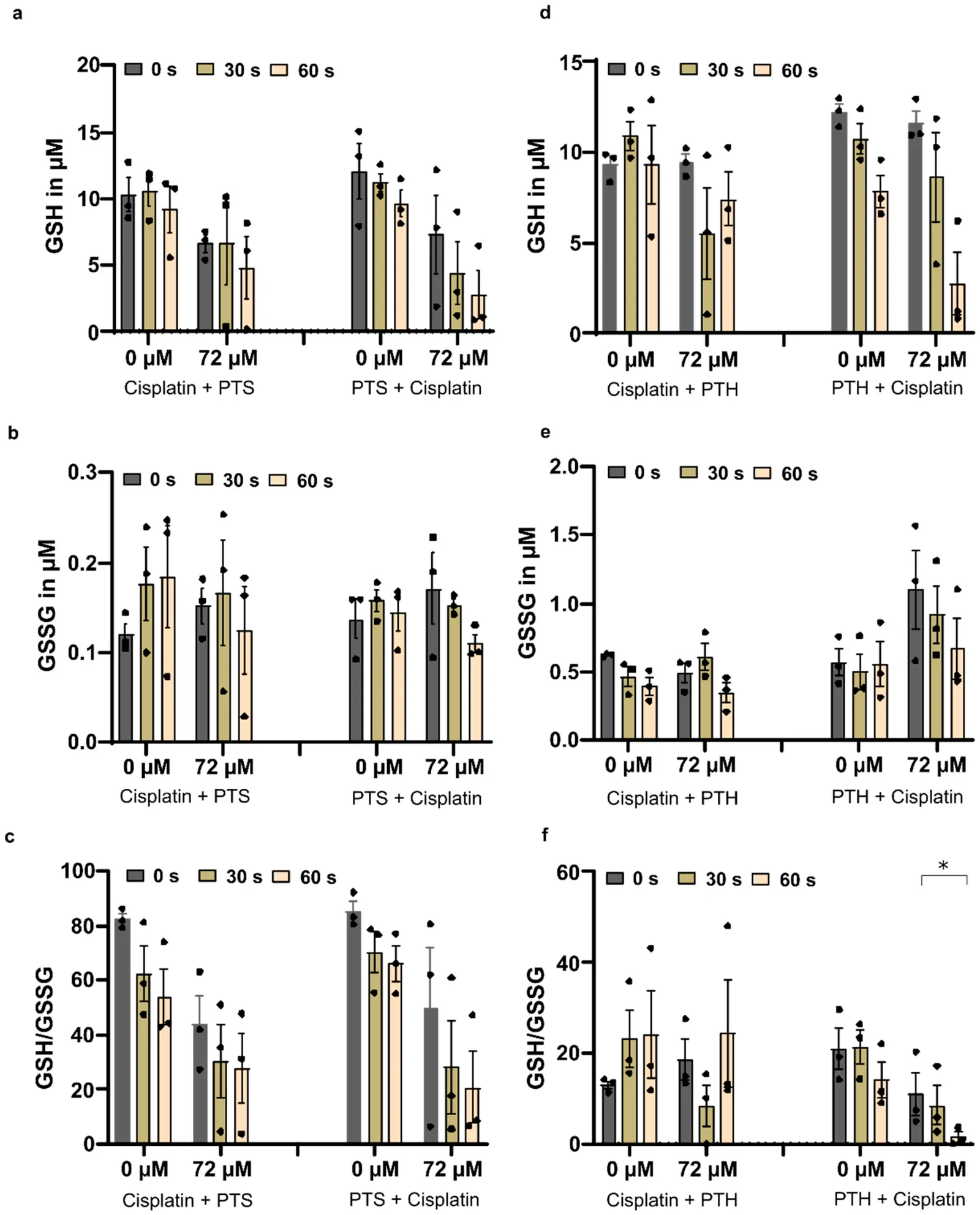
**Effect of combinatorial plasma treatment on cellular redox balance.** Both treatment sequences of the combined cisplatin + PTS/PTH treatment were evaluated for their impact on GSH and GSSG levels in SW954 cells. (**a**) Intracellular GSH concentration in SW954 cells following both treatment sequences of cisplatin and PTS. (**b**) Intracellular GSSG concentration in SW954 cells following both treatment sequences of cisplatin and PTS. (**c**) Calculated ratio of GSH/GSSG in μM from both PTS treatment sequences. (**d**) Intracellular GSH concentrations in SW954 cells following both treatment sequences of cisplatin and PTH. (**e**) Intracellular GSSG concentrations in SW954 cells following both treatment sequences of cisplatin and PTH. (**f**) Calculated ratio of GSH/GSSG in μM from both PTH treatment sequences. Data are shown as the mean ± SEM, *p < 0.05. Each data point represents one biological replicate.

For experiments involving multiple treatment groups (Fig. 2, Fig. 3, Fig. 4 and Fig. 5), statistical significance was assessed using two-way ANOVA. This was followed by Dunnett’s multiple comparison test comparing each cisplatin concentration to the corresponding 0 μM control within each ltAPD treatment time. For Fig. 5, the 0 s PTS/PTH condition was defined as the reference control, comparing each PTS/PTH treatment time within each cisplatin concentration for the respective multiple comparison analysis.

## 3 Results

### 3.1 RONS Dynamics and Cytotoxicity of PTS and PTH in Vulvar Carcinoma Cells

PTS and PTH were generated as depicted in Fig. 1a. Subsequently, the concentrations of hydrogen peroxide (H_2_O_2_) and nitrite (NO_2_^−^) were measured as surrogates representatively for the broad range of detectable reactive species presented in PTS and PTH to evaluate their oxidative potential. Therefore, different plasma treatment times and incubation durations were followed. Samples were generated using ltAPD exposure for 1, 3, or 5 min and analyzed immediately after treatment (0 h) and at multiple timepoints between 1 and 72 h (Fig. 1b–e and Fig. S1).

H_2_O_2_ concentrations in PTS increased proportionally with ltAPD treatment time, reaching 32.7 mg/L, 61.6 mg/L, and 92.5 mg/L after 1, 3, and 5 min, respectively (Fig. 1b,c, and Fig. S1A). In contrast, PTH samples showed consistently lower initial H_2_O_2_ levels (3.4 mg/L, 20.6 mg/L, and 41.7 mg/L at the same respective treatment times), indicating partial retention or limited release of RONS by the hydrogel matrix. Although H_2_O_2_ levels were lower in PTH across all conditions, a statistically significant difference between PTS and PTH was only observed at the 0 h timepoint, independently of the plasma treatment time. Over time, H_2_O_2_ concentrations in both systems decreased progressively. In PTS, levels declined steadily and became undetectable after 48 h regardless of initial treatment time. Interestingly, in PTH, H_2_O_2_ concentration slightly increased within the first hour post-treatment before similarly diminishing to near-zero levels by 48 h. This delayed release pattern may reflect matrix-associated generation or delayed diffusion of reactive species.

NO_2_^−^ concentrations followed similar treatment-time dependent trends. In PTS, immediately measured NO_2_^−^ levels were 4.4 mg/L, 16.7 mg/L, and 28.8 mg/L after 1, 3, and 5 min of ltAPD exposure, respectively (Fig. 1d,e and Fig. S1B). In comparison, NO_2_^−^ concentrations in PTH were significantly lower, amounting to 1.0 mg/L, 3.9 mg/L, and 7.7 mg/L across the same treatment times. While NO_2_^−^ levels in PTS remained relatively stable during the 72 h incubation period, NO_2_^−^ concentrations in PTH increased gradually over the first 4–6 h, reached a plateau, and then declined at later timepoints. These observations suggest that NO_2_^−^ in PTH may be released or formed over time within the hydrogel matrix, followed by degradation at longer intervals. Taken together, these data underscore distinct release kinetics and redox profiles of the two systems. PTS provides a high initial oxidative surge with rapid RONS decay, whereas PTH affords delayed and sustained delivery, particularly for NO_2_^−^. Despite differing relative concentrations, both systems show characteristic time- and dose-dependent patterns.

To assess potential pH-related effects, semi-quantitative pH measurements were performed in parallel with RONS analysis. Across all the plasma-treated conditions, pH values remained stable, with no evidence of progressive acidification or pH drift (Supplementary Fig. S2). Given the constant medium composition and absence of added acids or bases, these readings likely reflect method-dependent indicator responses than absolute bulk pH value. Nevertheless, the data suggest pH is unlikely to account for the observed effect on cytotoxicity. Next, to investigate whether the observed RONS profiles translated into cytotoxic effects, PTS and PTH were applied for 24 h to SW954 vulvar carcinoma cells, and cell viability was assessed (Fig. 1f,g). Both modalities demonstrated treatment-time-dependent reductions in cell viability. PTS exposure resulted in a marked cytotoxic effect, decreasing cell viability to 58% after 60 s of ltAPD treatment. Prolonged treatment further reduced viability to 7% at 120 s, with no significant additional reduction beyond this time point (Fig. 1f). Similarly, PTH formulations showed delayed cytotoxic effects compared to PTS. At 60 s, viability remained relatively high at 80% (Fig. 1g). However, with increasing treatment durations, viability declined sharply, reaching approximately 11% at 180 s. Near-complete cytotoxicity was achieved after 300 s for hydrogel-based plasma treatments.

### 3.2 Cytotoxic Effects of Cisplatin Alone and in Combination with PTS

To establish a baseline for cisplatin-induced cytotoxicity in SW954 cells, a dose-response analysis was conducted using a concentration range of 3–96 μM and incubation periods of 2, 4, 6, and 24 h (Supplementary Fig. S3). Minimal cytotoxicity was observed at lower concentrations (3–12 μM), irrespective of exposure time. Treatment with 24 μM and 48 μM reduced cell viability moderately to 84–93%. Notably, exposure to 96 μM cisplatin caused only a modest reduction in viability after 2 to 6 h (85–92%), whereas a decrease to 46% was observed following a 24-h incubation. These results confirm that both higher cisplatin concentrations and prolonged exposure are required to induce notable cytotoxic effects in vulvar carcinoma cells. To detect possible synergies of the combination treatment of cisplatin and ltAPD, previously evaluated cytotoxic as well as non-cytotoxic concentrations of cisplatin (12–96 μM) were applied. Since an incubation time between 2 to 6 h didn’t show major alterations, the 2 h time point was chosen opposing to the 24 h time point, which showed enhanced cytotoxicity.

Subsequently, the potential of PTS to enhance cisplatin efficacy in SW954 was evaluated using two treatment sequences (Fig. 2a). In the first sequence, cells were pre-treated with cisplatin for 2 h, followed by a 24 h PTS exposure. As shown in Fig. 2b, cisplatin alone (0 s PTS) produced minimal effects on viability, consistent with the earlier dose-response data. For instance, 96 μM cisplatin alone resulted in 86% viability. However, combining 96 μM cisplatin with 30 s PTS further reduced viability to 59%, indicating a potentiation of the cytotoxic effect. Although only one cisplatin-PTS combination yielded statistically significant reductions in viability, a trend toward increasing cytotoxicity was observed with longer PTS exposures and higher cisplatin doses. At 60 s PTS, even combinations with lower cisplatin concentrations showed enhanced cytotoxicity. This observed effect was supported by synergy analysis (Fig. 2c), where all combinations yielded positive synergy scores, although none exceeded the defined threshold of 20 for strong synergy. The highest synergy score in this sequence was 19.69, observed with the combination of 96 μM cisplatin and 60 s PTS.

In the second sequence, cells were pretreated with PTS for 2 h, followed by a 24 h cisplatin exposure (Fig. 2d). As expected, cisplatin-only controls (0 s PTS) showed a decreasing cell viability with increasing concentrations, the cellular effects after 24 h weren’t as pronounced as previously observed (Supplementary Fig. S3). Given the limited 2 h plasma exposure in this setup, PTS alone induced weaker cytotoxic effects compared to the first sequence. However, the overall cytotoxicity of the combination treatments in this sequence was greater than in the cisplatin-first sequence. Significant reductions in cell viability were observed for combinations of 30 s PTS with 72 μM and 96 μM cisplatin, for 60 s PTS combined with 24–96 μM cisplatin, and for 90 s PTS combined with 96 μM cisplatin. Corresponding synergy scores (Fig. 2e) were overall higher in this sequence than in the first, and several exceeded the synergy threshold of 20. The highest synergy score was observed with the combination of 96 μM cisplatin and 60 s PTS, indicating that pre-exposure to plasma more effectively sensitized the cells to cisplatin.

Together, these findings demonstrate that cisplatin alone induces moderate cytotoxicity in SW954 cells only at high concentrations and prolonged incubation. However, co-treatment with PTS enhances its efficacy, especially at previously observed non-cytotoxic concentrations. Moreover, the treatment sequence plays a critical role, plasma pre-exposure elicits greater chemosensitization than the reverse order, supporting a redox-priming mechanism.

### 3.3 Cytotoxicity and Synergy of Cisplatin Combined with PTH

To evaluate the potential of PTH in enhancing cisplatin efficacy, combination treatments were performed using both treatment sequences (Fig. 3a). These experiments were designed to mirror the PTS combination assays, using identical cisplatin concentrations but longer plasma exposure durations, since PTH previously showed delayed cytotoxic effects.

In the first sequence, cells were treated with cisplatin for 2 h, followed by PTH exposure for 24 h (Fig. 3b). Notably, the co-application of 30 s PTH with increasing cisplatin concentrations resulted in a clear synergistic effect. Even at 24 μM cisplatin, synergy scores exceeded the synergy threshold (>20), indicating that lower drug concentrations could be potentiated by brief plasma-hydrogel exposure. Stronger synergistic responses were observed with 48–96 μM cisplatin, particularly in combination with 30 s PTH. The highest synergy score (32.74) was achieved with 48 μM cisplatin and 30 s PTH, reflecting both a statistically significant and biologically relevant reduction in cell viability. Although synergy persisted with 60 s PTH treatment, the effect was slightly less pronounced compared to the 30 s condition. At 90 s PTH, the combination treatment reached a viability plateau around 20%, and no further significant reduction was observed with increasing cisplatin concentrations and longer PTH treatment times. In fact, longer PTH exposures combined with higher cisplatin doses began to yield negative synergy scores, suggesting a saturation at prolonged plasma exposure (Fig. 3c).

In the reverse treatment sequence, PTH was administered for 2 h prior to a 24 h cisplatin exposure (Fig. 3d). Contrary to the previous results, this sequence did not produce robust synergy (Fig. 3e). Although viability reductions were observed, synergy scores remained below the significance threshold. The highest score reached only 10.78, and synergy was not consistently associated with increased plasma treatment duration or higher drug concentrations. Interestingly, cisplatin alone produced stronger cytotoxicity in these experiments than previously observed, potentially masking the synergistic benefit of plasma preconditioning. Furthermore, 72 μM cisplatin appeared more effective than 96 μM in this assay, suggesting possible variability in response, depending on exposure context.

Taken together, these findings contrast with the observations from the PTS combination experiments. While PTS-based synergy was stronger in the plasma-first sequence, PTH showed the opposite trend. Specifically, cisplatin followed by PTH produced more substantial and synergistic cytotoxic effects than the reverse order. This suggests that the timing of plasma-hydrogel application is critical and may modulate the availability or activity of RONS at the time of drug exposure. These differences highlight the importance of delivery kinetics and treatment order in optimizing plasma-drug combinatorial regimens.

### 3.4 Caspase-3/7 Activation Following Cisplatin and Plasma Treatment Combinations

To determine whether the combined application of plasma treatment and cisplatin promotes apoptosis in vulvar carcinoma cells, caspase-3/7 activity was measured following sequential administration of PTS or PTH with cisplatin. Both treatment sequences, plasma after cisplatin (Fig. 4a,c) and plasma before cisplatin (Fig. 4b,d) were evaluated.

In the absence of plasma exposure (0 s PTS/PTH), increasing concentrations of cisplatin alone did not significantly elevate caspase-3/7 activity (Fig. 4a–d).

However, plasma treatment alone (30 s PTS) induced a measurable increase in caspase activity, which was further amplified in a cisplatin dose-dependent manner. In the first sequence (Fig. 4a) this effect was enhanced following 60 s of PTS exposure, where caspase-3/7 activity increased in parallel with higher cisplatin concentrations. A similar sequence- and dose-dependent trend was observed with PTH (Fig. 4c,d). Caspase-3/7 activity increased with longer plasma activation times and higher cisplatin doses, regardless of whether plasma preceded or followed cisplatin treatment.

Taken together, these findings demonstrate that plasma exposure, delivered either as PTS or PTH, markedly enhances apoptotic signaling in vulvar cancer cells in a time- and dose-dependent manner. Importantly, the fact that cisplatin alone failed to trigger detectable apoptosis, whereas plasma substantially augmented caspase-3/7 activation in combination conditions, underscores plasma-mediated redox disruption as a key upstream driver of apoptotic susceptibility in this model. Reversing the sequence yielded a comparable but even more consistent pattern. When PTS was applied prior to cisplatin administration (Fig. 4b), both 30 s and 60 s plasma exposures sensitized SW954 cells to subsequent cisplatin-induced apoptosis.

### 3.5 Effect of Combinatorial Plasma Treatment on Cellular Redox Balance

To investigate how the sequence of plasma treatment and cisplatin influences intracellular oxidative stress, we quantified the glutathione redox couple (GSH/GSSG), following treatment with PTS or PTH in combination with cisplatin. A cisplatin concentration of 72 μM was selected based on previous experiments demonstrating a robust cellular response when combined with PTS or PTH.

In both sequences using PTS (Fig. 5a–c), treatment with 72 μM cisplatin alone reduced the GSH/GSSG ratio compared to untreated controls (Fig. 5c), indicating a shift toward a more oxidized intracellular state. This effect was further enhanced when PTS was added. Notably, the reduction in the GSH/GSSG ratio was more pronounced when PTS preceded cisplatin exposure, suggesting that plasma pre-treatment more effectively compromises the redox buffering capacity of the cell, potentially sensitizing it to subsequent drug-induced oxidative damage. A similar trend was observed with PTH (Fig. 5d–f). Application of PTH prior to cisplatin equally resulted in a lower GSH/GSSG ratio compared to the cisplatin first sequence, consistent with greater oxidative stress (Fig. 5f).

Collectively, these results indicate that both plasma modalities, PTS and PTH, exert redox-disruptive effects that are significantly influenced by treatment sequence. Pre-treatment with plasma consistently led to a more oxidized cellular redox state at the time of cisplatin exposure, aligning with the observed enhancement in cytotoxicity and synergistic interaction for PTS. Taken together, the results from the caspase-3/7 assay and the GSH/GSSG analysis of the PTH-first sequence show a strong cellular response and equally suggest a cellular cytotoxic priming toward cisplatin exposure, like in the PTS sequence, even though these findings are not reflected in the MTS results. The hypothesis that high cisplatin cytotoxicity masked the observed effects was taken into account for subsequent data interpretation.

## 4 Discussion

Despite advances in surgical management and chemoradiation, treatment outcomes for advanced or recurrent vulvar carcinoma remain limited, and therapeutic diversification is often constrained by drug toxicity rather than lack of efficacy [34]. These limitations highlight the need for new approaches that enhance tumor cell susceptibility to existing agents without increasing systemic burden. ltAPD has emerged as a promising modality in this regard, as its biological effects are mediated by redox-active species that can perturb cellular homeostasis through mechanisms orthogonal to conventional chemotherapeutic drugs [14]. Thus, currently plasma-based interventions are increasingly considered as sensitizers capable of reprogramming the redox state of tumor cells, thereby amplifying the efficacy of standard agents at equal or even reduced doses [27,35]. Hence, evaluating plasma-drug combinations as a potential therapy regimen and their treatment order is of high translational relevance.

The present study provides a comparative analysis of two plasma-derived therapeutic modalities (PTS and PTH), and demonstrates how their relative RONS delivery profiles give rise to differential biological effects and combinatorial efficacy with cisplatin in SW954, HPV independent immortalized vulvar cancer cell line. Although both formulations originate from the same plasma source and contain comparable classes of oxidants, their physicochemical properties determine different interaction kinetics with the cellular microenvironment. These differences in RONS availability, persistence, and spatial confinement translate into distinct cytotoxic, apoptotic, and redox-modulating outcomes, underscoring that the choice and sequencing of plasma modality are critical variables in the rational design of plasma-enabled cancer therapies.

The substantially higher H_2_O_2_ and NO_2_^−^ concentrations detected in PTS immediately after plasma activation indicate that reactive species accumulate freely in the liquid phase. In contrast, the hydrogel matrix in PTH restricts diffusion, resulting in a slower and more prolonged release of RONS. This fundamental difference is important when choosing the appropriate plasma formulation for a specific biological application. PTS, therefore, could act as a fast, high-flux delivery system that generates rapid oxidative stress [36,37,38], whereas PTH likely functions as a controlled-release depot that provides spatially confined and sustained exposure [39,40]. Potential variability in probe-to-surface distance may influence plasma exposure and, consequently, the chemical composition of PTS. However, the precise APC E1 mode used in this study is designed to deliver low-energy, superficial plasma effects with minimal thermal penetration. According to the manufacturer, the resulting tissue effect is largely independent of probe-tissue distance within the recommended operating range [41]. Therefore, minor variations in probe positioning are unlikely to have substantially affected the chemical consistency of PTS or PTH under the applied experimental conditions.

The relative stability of nitrite also suggests that long-lived RONS from either modality may maintain a low-level oxidative environment capable of influencing redox signaling and cellular decision pathways beyond the initial activation period [42]. These differences in delivery kinetics have direct biological consequences. A rapid oxidative burst, as seen with PTS, can overwhelm cellular antioxidant defenses and trigger acute stress responses, membrane damage, and early cytotoxicity [38]. In contrast, the gradual oxidative input delivered by PTH is more likely to shift intracellular redox balance over time, deplete glutathione, and weaken adaptive stress responses [39]. This slower redox modulation may not cause immediate cell death, but it can sensitize tumor cells to additional treatments such as DNA-damaging agents.

As monotherapies, both PTS and PTH reduced SW954 cell viability in a treatment time-dependent manner. PTS produced comparable cytotoxicity at shorter activation times, consistent with its higher immediate oxidative burden. Notably, PTH still induced significant cell death despite its lower measurable levels of freely diffusible RONS. This suggests that additional reactive intermediates, retained within the hydrogel, contribute to its biological activity [40]. This interpretation is consistent with reports showing that hydrogels can generate long-lived oxidants or matrix-bound reactive intermediates, which support localized oxidative reactions at the cell-hydrogel interface even when extracellular RONS levels are low. Such matrix-associated reactivity helps explain how PTH maintains cytotoxic potential despite delivering a lower apparent oxidative load to the surrounding medium [40,43,44]. Furthermore, the presence of a DMEM overlay above the hydrogel introduces an additional diffusion layer that may influence the transport and effective concentration of plasma-derived reactive species. This liquid phase can contribute to dilution and delayed diffusion of RONS toward the cell layer, thereby reducing peak concentrations at the cellular interface. While the porous and highly hydrated structure of the hydrogel itself is unlikely to impose a strong diffusion barrier for small reactive species, it may act as a transient reservoir that modulates release kinetics [45]. Together, these effects likely contribute to the delayed and attenuated biological responses observed for PTH compared to PTS.

A key potential translational finding of this study is the strong synergistic interaction between plasma exposure and cisplatin treatment. Synergistic effects were evaluated using the ZIP model, with scores above 20 considered indicative of strong synergy. Importantly, synergy was interpreted in the context of both statistical and biological relevance, based on comparisons between monotherapy and combination treatments. Under PTS conditions, plasma pre-treatment markedly enhanced cisplatin efficacy, indicating that plasma does not simply act additively but actively renders tumor cells more vulnerable to the drug. The differential cytotoxic and synergistic effects observed between PTS and PTH can be attributed to their distinct RONS delivery kinetics. While PTS induces a rapid and high initial burst of reactive species, PTH provides a delayed and sustained release due to partial retention and gradual diffusion within the hydrogel matrix. This difference is particularly relevant in combinatorial settings, where the timing of oxidative stress relative to chemotherapeutic exposure is critical.

The reduced or absent synergistic effects observed for PTH under certain conditions likely reflect suboptimal temporal overlap between peak RONS availability and cisplatin activity. In addition, prolonged RONS exposure may lead to saturation effects or overlapping cytotoxic mechanisms, resulting in elevated baseline cytotoxicity that diminishes the apparent synergistic interaction. In contrast, the immediate oxidative burst induced by PTS may more effectively prime tumor cells for subsequent chemotherapy. These findings highlight the importance of temporal control of RONS delivery and suggest the existence of an optimal therapeutic window, in which controlled and appropriately timed RONS exposure maximizes chemo-sensitization without inducing excessive baseline damage. Accordingly, further optimization of hydrogel composition, including tuning of RONS retention, diffusion behavior, and interaction with the cellular microenvironment, may enhance the therapeutic potential of PTH-based approaches. The superiority of the plasma-first sequence supports a priming mechanism in which plasma-induced oxidative imbalance compromises cellular stress-response capacity, thereby intensifying the effects of subsequent DNA damage with cisplatin. This interpretation is supported by the increased caspase-3/7 activity and the pronounced decrease in the GSH/GSSG ratio observed under combined treatment, both indicating reduced redox buffering at the time of drug exposure. Importantly, within the validated exposure window, cisplatin alone did not induce detectable apoptosis in SW954 cells, whereas both PTS and PTH significantly increased caspase-3/7 activity. This distinction highlights plasma-mediated redox perturbation as a key upstream trigger of apoptotic activation in this model. Although PTH priming was not as clearly reflected in the cytotoxicity assays, it produced strong effects in the redox and apoptotic pathway analyses, indicating that PTH also induces a priming state, but with reduced intensity compared to PTS. Additionally, the observed discrepancy between redox and apoptotic responses and MTS-based viability in the PTH-first condition warrants consideration. While significant alterations in GSH/GSSG ratios and increased caspase-3/7 activity indicate early oxidative stress and apoptotic signaling, these changes may not immediately translate into detectable reductions in metabolic activity of PTH as measured by MTS assay and their corresponding synergies. This divergence likely reflects differences in assay sensitivity and the temporal dynamics of cellular responses. In particular, the delayed and sustained release of RONS from PTH may induce temporally shifted biological effects, where early redox perturbation and apoptotic priming precede measurable losses of metabolic viability. These findings highlight the importance of considering assay-specific readouts and timing when interpreting combinatorial treatment effects. Among the underlying mechanisms, glutathione depletion emerges as particularly relevant, given the central role of GSH in mediating cisplatin resistance through conjugation, detoxification, and maintenance of redox signaling homeostasis [46]. Pre-treatment with plasma resulted in a greater reduction in the GSH/GSSG ratio than when plasma followed cisplatin exposure, indicating a more pronounced oxidative shift when the redox challenge preceded chemotherapy. This sequence-dependent effect suggests that plasma-induced oxidative stress compromises the cells antioxidant buffering system, thereby diminishing its capacity to neutralize reactive intermediates and withstand subsequent cisplatin-induced damage. In contrast, when cisplatin was administered first, the magnitude of redox disruption was notably reduced, likely reflecting partial activation of glutathione biosynthesis and stress adaptation pathways prior to plasma application. The dynamics of reduced (GSH) and oxidized (GSSG) glutathione levels further support this interpretation. In the plasma-first sequence, GSH levels declined progressively with increasing plasma exposure, while GSSG levels remained elevated or declined more slowly, reflecting ongoing oxidation of the glutathione pool. The resulting sharp drop in the GSH/GSSG ratio marks a critical loss of redox buffering capacity at the time of cisplatin challenge. By contrast, the cisplatin-first sequence preserved higher GSH/GSSG ratios, consistent with more effective redox compensation [47].

In addition to glutathione depletion, plasma-derived RONS are known to induce multiple interconnected cellular stress responses that may contribute to enhanced sensitivity to cisplatin. These include mitochondrial dysfunction, characterized by loss of membrane potential and increased mitochondrial ROS production, as well as the induction of DNA damage through oxidative base modifications and strand breaks ([14,48]). Furthermore, reactive species can modulate redox-sensitive signaling pathways, including MAPK and NF-κB, thereby influencing cell cycle regulation, stress responses, and apoptotic signaling [49,50]. Collectively, these processes may act together to promote apoptotic priming, lower the threshold for chemotherapy-induced cell death, and reduce cellular resilience to cytotoxic stress. Such multi-level redox perturbation likely underlies the observed enhancement of cisplatin efficacy following plasma treatment.

These findings also have direct implications for chemoresistance. Elevated intracellular GSH is a canonical mechanism of cisplatin resistance, supporting both drug detoxification and buffering of treatment-induced oxidative stress [46,51]. By depleting GSH and shifting the GSH/GSSG equilibrium toward an oxidized state, plasma treatment destabilizes this defense axis, lowers the apoptotic threshold, and reinstates susceptibility to stress-induced cell death. Plasma, therefore, acts as a redox-conditioning agent that re-sensitizes tumor cells to cisplatin. This mechanistic complementarity provides a rationale for integrating plasma-based redox modulation into regimens for cisplatin-refractory disease.

The translational relevance further extends to vulvar intraepithelial neoplasia (VIN), the premalignant precursor of vulvar carcinoma. High-grade VIN, particularly HPV-independent lesions, often display redox imbalance and limited therapeutic responsiveness, while management is constrained by the need to preserve tissue integrity and function [6,52]. Plasma-based approaches, especially PTH, enabling spatially confined and temporally sustained delivery, could represent a plausible tissue-sparing adjunct in this setting. By reprogramming redox signaling and enhancing chemosensitivity, ltAPDcould serve as a minimally invasive alternative or complement to current treatment options, particularly in recurrent or multifocal VIN.

The two delivery platforms studied here serve distinct clinical niches. PTS is suitable for superficial or lavage-based applications (e.g., intraoperative irrigation of resection sites), where transient but high-intense oxidative exposure is desirable. By contrast, PTH enables anatomically confined, sustained dosing, appropriate for lesions or postoperative sites adjacent to radiosensitive or functionally critical tissue. From a translational perspective, the distinct properties of PTS and PTH may inform different clinical implementation strategies. While PTS may be particularly suited for rapid tumor preconditioning prior to chemotherapy, PTH offers additional versatility as a localized delivery system. Given the demonstrated synergistic and additive effects of plasma with cisplatin, administration during active chemotherapy may be preferable to maximize therapeutic efficacy. Notably, even in conditions where strong synergy was not observed for PTH, no antagonistic effects were detected, suggesting that concurrent use is unlikely to compromise treatment outcomes. Furthermore, the plasma generated hydrogel platform may serve as a multifunctional delivery vehicle for additional therapeutic agents, including emerging strategies such as immune-modulatory compounds or immune checkpoint inhibitors, as proposed in recent studies allowing a precisely controlled release [53,54,55]. Together, these considerations highlight the potential of plasma-based therapies, particularly in combination with advanced biomaterial platforms, to be integrated into multimodal cancer treatment regimens.

This study holds some limitations that should be considered when interpreting the findings and for future outlook. All experiments were performed in a single VSCC cell line (SW954), which does not completely capture the biological heterogeneity of vulvar cancer, including HPV-associated and HPV-independent subtypes. In addition, the absence of non-malignant epithelial controls limits the assessment of treatment selectivity and therapeutic window. Future studies incorporating multiple cancer models, non-malignant systems, and more advanced three-dimensional or patient-derived models are needed to enhance translational relevance. Also, the physicochemical characterization of the PTH was limited. The present work focused primarily on functional evaluation of RONS and biological activity, whereas detailed structural and rheological analyses of the hydrogel matrix were not performed. Such characterization would be valuable for understanding material properties, diffusion behavior, and their impact on therapeutic efficacy.

Next, the quantification of RONS was based on semi-quantitative test strip methods. H_2_O_2_ and NO_2_^−^ values should be considered semi-quantitative upper-bound estimates, likely influenced by limitations of test strip-based readouts, rather than exact equilibrium concentrations. Although the observed biological responses are consistent with increased oxidative stress, further validation using quantitative, matrix-adapted analytical approaches is warranted. Finally, the mechanistic conclusions are primarily supported by indirect indicators of redox modulation, including changes in the GSH/GSSG ratio and caspase-3/7 activity. While these findings are consistent with plasma-induced oxidative stress and apoptotic priming, additional studies employing targeted mechanistic approaches, such as reactive species scavenging or pathway-specific inhibition, will be required to establish causal relationships. Despite these limitations, the present study provides a systematic comparison of plasma delivery modalities and demonstrates their potential to enhance cisplatin efficacy through redox-associated mechanisms.

## 5 Conclusion

**Figure 6:**
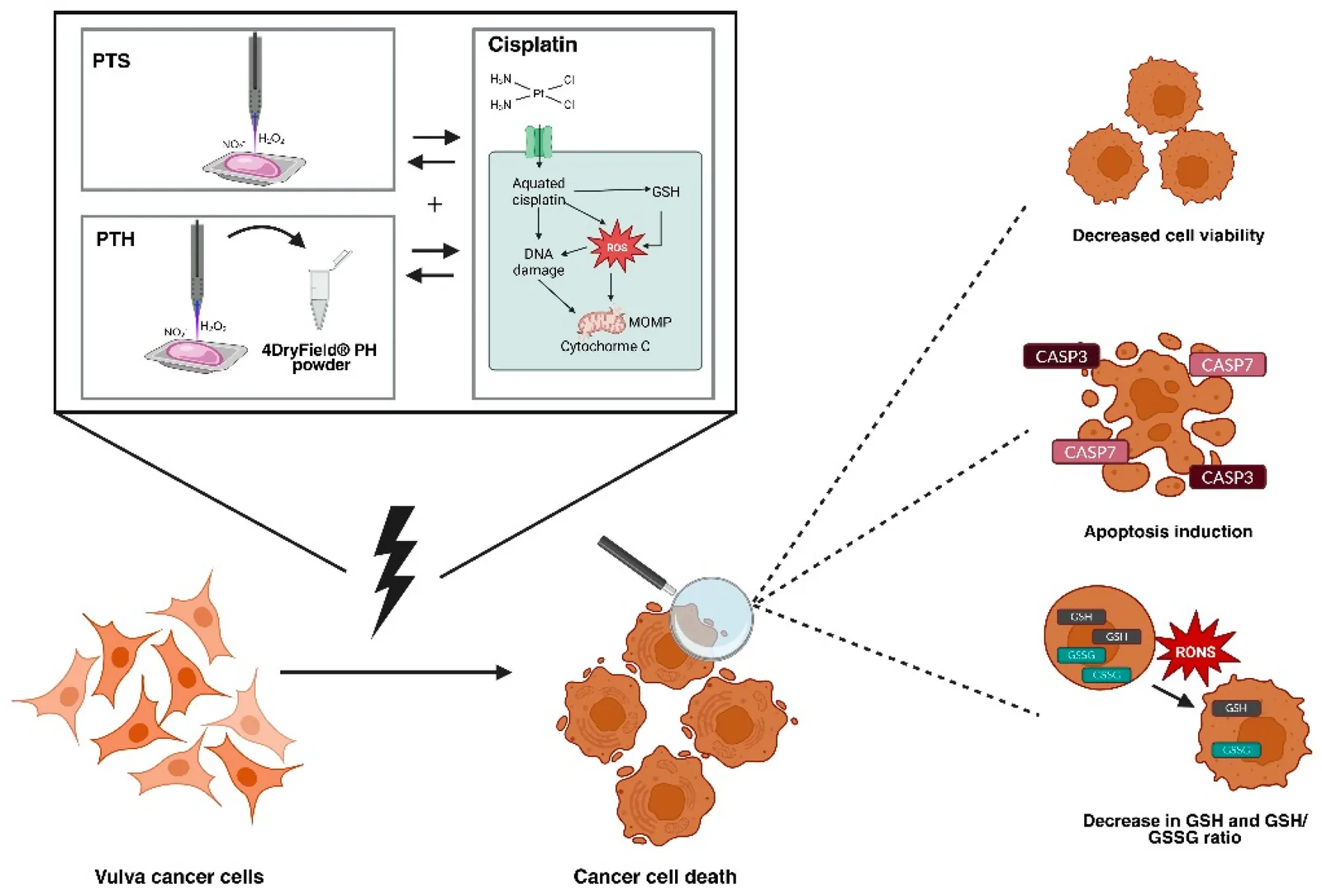
**Plasma-mediated combinatorial therapy and its downstream effects in the SW954 vulvar cancer cell line.** Treatment with PTS and PTH in combination with cisplatin significantly reduced cell viability and enhanced apoptosis induction compared with cisplatin monotherapy. Furthermore, the plasma-primed combinatorial approach markedly decreased the GSH/GSSG ratio in SW954 cells, confirming enhanced oxidative stress and disruption of cellular redox homeostasis as a key mechanism underlying the improved therapeutic efficacy.

Taken together, both the studied modalities with the plasma primed approach combined with cisplatin showed reduced cell viability and apoptosis induction. In addition, it rendered a decrease in GSH/GSSG ratio disruption of cellular redox homeostasis in SW954 cells (Fig. 6). The present findings indicate PTS and PTH as plasma-based strategies that offer distinct advantages for plasma-mediated combination therapy. The ability of plasma exposure to induce redox priming provides a mechanistic rationale for lowering cisplatin doses without compromising therapeutic efficacy, thereby reducing systemic toxicity. Moreover, the capacity for controlled and spatially restricted delivery, particularly through PTH, suggests a potential role for plasma formulations as tissue-sparing adjuncts in anatomically sensitive settings. This is especially relevant for HPV-independent high-grade VIN, where preservation of vulvar structure and function is a major clinical priority. By selectively reprogramming redox signaling and restoring chemosensitivity, plasma-enabled approaches may therefore complement or enhance existing treatment regimens. Overall, these results support the development of plasma-derived therapeutic modalities as redox-guided combination strategies for the management of vulvar precancerous lesions and cancer.

## Data Availability

All data supporting the results of this study are included within the article and its Supplementary Materials.

## Supplementary Materials

The supplementary material is available online at https://www.techscience.com/doi/10.32604/or.2026.081755/s1.

## Abbreviations

BCC	Basal Cell Carcinoma
dVIN	Differentiated Vulvar Intraepithelial Neoplasia
DPBS	Dulbecco’s Phosphate Buffered Saline
GSH	Glutathione
GSSG	Oxidized glutathione
H_2_O_2_	Hydrogen peroxide
HPV	Human Papilloma Virus
HSIL	High-grade squamous intraepithelial lesions
LSIL	Low-grade squamous intraepithelial lesions
ltAPD	Low-thermal argon plasma devitalization
NO_2_	Nitrogen dioxide
PTH	Plasma treated hydrogels
PTS	Plasma treated solution
RONS	Reactive oxygen and nitrogen species
ROS	Reactive oxygen species
SEM	Standard Error Mean
VIN	Vulvar intraepithelial neoplasia
VSCC	Vulvar squamous cell carcinoma
ZIP	Zero Interaction Potency
