# Supplementary material for: Combinatorial Effects of Plasma-Treated Solution and Plasma-Treated Hydrogel with Cisplatin in Vulvar Cancer Cells
Source: Oncol Res. 2026 Aug 13;34(9):1. doi: 10.32604/or.2026.081755 (PMC13494491; doi:10.32604/or.2026.081755)
Supplement: Supplementary file 1 [file OncolRes-34-81755-s001.zip › TSP_OR_81755-Supplementary_Files.pdf]

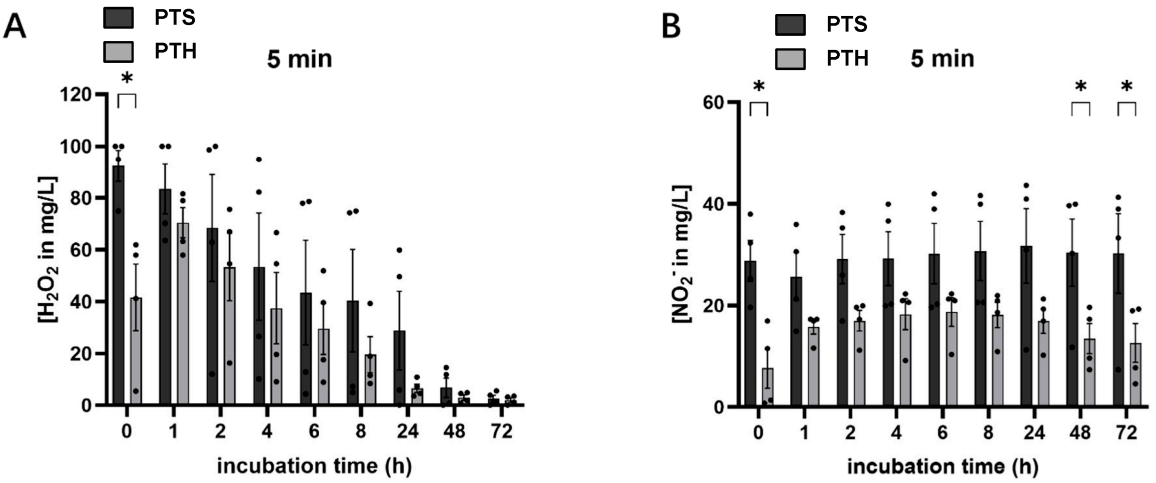

Figure S1: RONS dynamics in Plasma Treated Solution (PTS) and Plasma Treated Hydrogel (PTH) treatment modalities. (**A**) Hydrogen peroxide (H_2O_2) concentrations were measured immediately after plasma treatment (0 h) and following incubation periods ranging from 1–72 h for 5 min of plasma treatment. (**B**) Nitrite (NO_2^-) concentrations were measured immediately after treatment (0 h) and after incubation for 1–72 h for 5 min of plasma treatment. Data are shown as the mean ± SEM, *$p < 0.05$. Each data point represents one biological replicate.

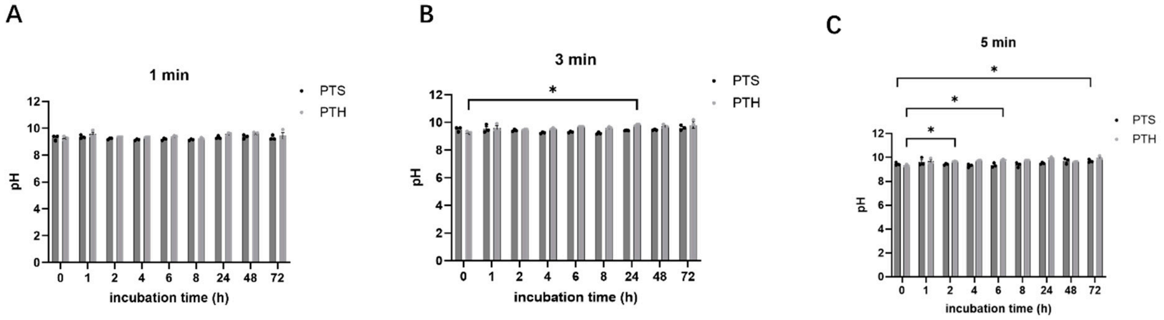

Figure S2: pH dynamics in PTS and PTH treatment modalities. (**A**) pH levels were measured immediately after plasma treatment (0 h) and following incubation periods ranging from 1–72 h for 1 min of plasma treatment. (**B**) pH levels were measured immediately after plasma treatment (0 h) and following incubation periods ranging from 1–72 h for 3 min of plasma treatment. (**C**) pH levels were measured immediately after plasma treatment (0 h) and following incubation periods ranging from 1–72 h for 5 min of plasma treatment. Data are shown as the mean ± SEM, *$p < 0.05$. Each data point represents one biological replicate.

Copyright © 2026 The Author(s). Published by Tech Science Press. This work is licensed under a Creative Commons Attribution 4.0 International License, which permits unrestricted use, distribution, and reproduction in any medium, provided the original work is properly cited.

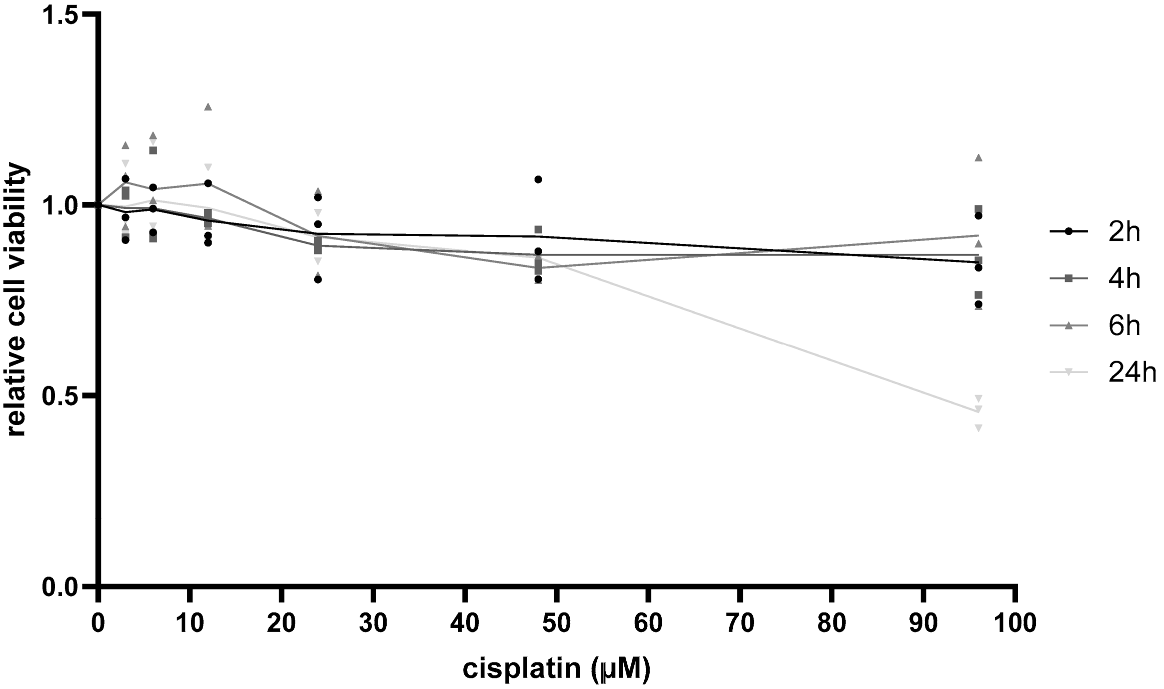

Figure S3: Dose—response curve of cisplatin treated on SW954 vulvar cancer cells. SW954 vulvar cancer cells were treated with increasing concentrations of cisplatin (3–96 µM) to assess dose-dependent effects on cell viability. Cells were incubated for 2, 4, 6, and 24 h to evaluate time-dependent responses. Cell viability was measured using MTS assay. Data are shown as the mean ± SEM. Each data point represents one biological replicate.
